# Dataset on soil microbial community composition based on 16S rRNA gene OTUs from estuarine wetlands in China

**DOI:** 10.1016/j.dib.2025.112345

**Published:** 2025-12-02

**Authors:** Yingjie Ma, Shuhan Wang, Jiale Ding, Yingpai Liu, Yonghui Wang, Zongxiao Zhang

**Affiliations:** a College of Geography Science and Tourism, Xinjiang Normal University, Urumqi, 830054, China; b Technical Research Center for Environmental Geotechnical Engineering Restoration and Resource Utilization, Xinjiang Normal University, Urumqi 830054, China

**Keywords:** Microbial community, Molecular ecological network, Network topology, Keystone taxa

## Abstract

This dataset comprises molecular ecological networks (MENs) and associated microbial community data from surface soils (0–5 cm depth) across nine Chinese estuarine wetlands: Liaohe Estuary (Liaoning province), Haihe Estuary (Tianjin Municipality), Yellow River Estuary (Shandong province), Sheyanghe Estuary (Jiangsu province), Changjiang Estuary (Shanghai Municipality), Oujiang Estuary (Zhejiang province), Jiulongjiang Estuary (Fujian province), Zhujiang Estuary (Guangdong province), and Beibuwan Gulf (Guangxi province). Twenty-nine composite samples (15–20 subsamples/site) were collected during August-September 2019.

Data include: Bacterial (16S V4-V5) and fungal (ITS1) OTU tables clustered at 97 % similarity (SILVA/UNITE databases; NCBI SRA PRJNA755846); Global and regional MEN adjacency matrices (Northern/Eastern/Southern China Sea groupings); Network topology indices (node/edge counts, average degree, modularity); Node-level metrics (betweenness centrality, clustering coefficient); Identified "Broker'' OTUs based on network indexes for individual nodes.

MENs were constructed using the Molecular Ecological Network Analysis Pipeline (MENAP, http://ieg2.ou.edu/MENA/) with Pearson correlations of log-transformed OTU abundances. Only OTUs present in >50 % of samples per group were included, with significance thresholds determined via Random Matrix Theory.

Specifications Table

**Table:** 

Subject	Biology
Specific subject area	Microbial ecology, Coastal Microbial Ecology, Molecular ecological network
Type of data	Table, Image, Analyzed
Data collection	Surface soil samples (0–5 cm) were collected as representative composites (15–20 subsamples/area) from 2–4 areas across nine Chinese estuaries. Samples for DNA analysis were preserved in liquid nitrogen. Total genomic DNA was extracted using FastDNA SPIN Kit for Soil (MP Biomedicals). Bacterial 16S rRNA V3-V4 regions (primers 338F/806R) and fungal ITS1 regions (primers ITS1737/ITS2043) were amplified. Libraries prepared with NEXTflex™ Rapid DNA-Seq Kit were sequenced on Illumina MiSeq PE250 platform (2 × 250 bp). Raw reads processed via FLASQ v0.14.1 were clustered into OTUs (97 % similarity, UPARSE) and taxonomically classified against SILVA (bacteria) and UNITE (fungi) databases.
Data source location	Surface soils were collected from intertidal zones of nine estuaries along China's coastline: Northern China Sea: Liaohe Estuary (Latitude range: 40°48′N-40°54′N, Longitude range: 121°49′E), Haihe Estuary (Latitude range: 38°46′N-39°50′N, Longitude range: 117°34′E-117°50′E), Yellow River Estuary (Latitude range: 37°49′N-38°51′N, Longitude range: 118°12′E-119°15′E). Eastern China Sea: Sheyanghe Estuary (Latitude range: 32°01′N-33°28′N, Longitude range: 120°18′E-120°36′E), Changjiang Estuary (Latitude range: 30°16′N-30°48′N, Longitude range: 121°27′E-121°58′E), Oujiang Estuary (Latitude range: 27°49′N-28°13′N, Longitude range: 120°44′E-120°51′E). Southern China Sea: Jiulongjiang Estuary (Latitude range: 24°27′N-24°46′N, Longitude range: 117°35′E-118°42′E), Zhujiang Estuary (Latitude range: 22°19′N-22°44′N, Longitude range: 113°00′E-113°46′E), Beibuwan Gulf (Latitude range: 21°41′N-21°42′N, Longitude range: 108°19′E-109°17′E).
Data accessibility	Repository name: Dataset on soil microbial community composition based on 16S rRNA gene OTUs from estuarine wetlands in China. Data identification number: 10.5281/zenodo.16585119 Direct URL to data: https://doi.org/10.5281/zenodo.16585119
Related research article	None.

## 1 Value of the Data

• The dataset captures microbial interaction networks and physicochemical properties from 29 soil samples across 9 major estuaries spanning [1].
• Catalogs high-connectivity "Broker'' OTUs with taxonomic and topological attributes, aiding research on microbial roles in ecosystem functions.
• Includes pre-constructed bacterial/fungal interaction networks (global + regional scales) with topology files (nodes/edges) and key metrics (connectivity, modularity), enabling direct reuse for modeling or comparative studies [2].
• Combines sequencing-derived microbial data with 12 physicochemical measurements (e.g., iron, nitrate, organic carbon) per sample, supporting analyses of soil-microbe relationships.

## 2 Background

Estuarine wetlands, as unique coastal ecosystems, play pivotal roles in biogeochemical cycling, with soil microbial communities being key drivers of these processes [3]. Understanding microbial interactions through molecular ecological networks (MENs) is crucial for elucidating community assembly and functional dynamics. However, large-scale comparative studies on MENs across geographically distinct estuarine wetlands in China remain limited.

This dataset was compiled to address this gap, focusing on nine major estuaries spanning the Northern, Eastern, and Southern China Seas. Methodologically, it builds on 16S rRNA V3-V4 and ITS1 region sequencing, coupled with MEN construction via the Molecular Ecological Network Analysis Pipeline (MENAP) and Random Matrix Theory-based thresholding, to capture microbial co-occurrence patterns and topological characteristics systematically [4]. The data aim to provide a foundational resource for exploring regional differences in estuarine soil microbial networks.

## 3 Data Description

**Fig. 1:**
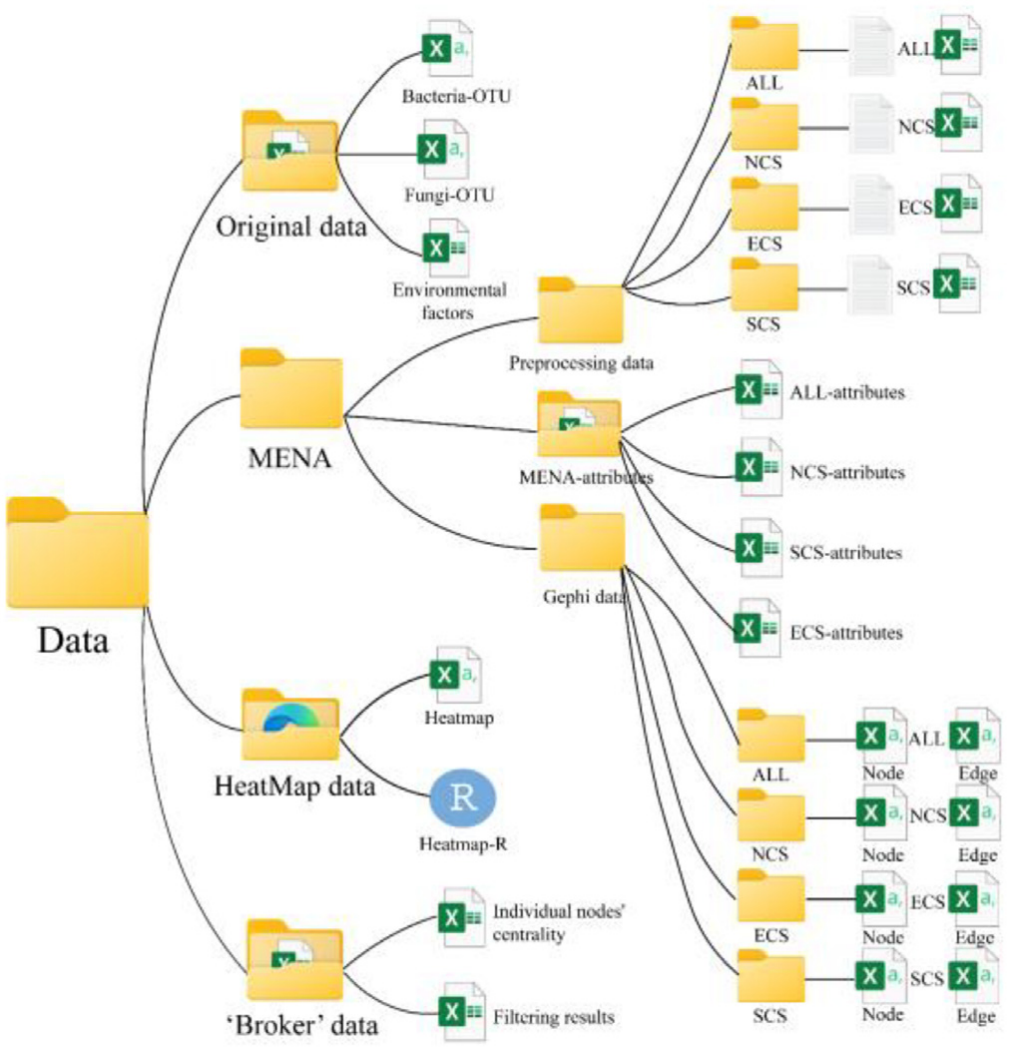
All data folders.

The dataset is systematically organized into four primary directories, each containing specific subfolders and files for raw and analyzed data. The hierarchical structure (Fig. 1) and detailed file descriptions (Table 1) enable straightforward navigation and reproducibility.

**Table 1:** Description of each field of the collected data.

Field		Description
Original data	Bacteria-OTU	The OTU table of bacteria at all sampling points
	Fungi-OTU	The OTU table of fungi at all sampling points
	Environmental factors	Tables of environmental factors at all sampling points
MENA	Preprocessing data	The OTU dataset after grouping based on the spatial distribution of sampling areas
	MENA-attributes	The various attributes of the results after going through the MENA platform
	Gephi data	The node and edge data of each group of networks after going through the MENA platform
HeatMap data	Heatmap	Heatmap's OTU dataset, grouping, and attribute table
	Heatmap-R	R language code for heatmap
‘Broker’ data	Individual nodes' centrality	The numerical value of the Individual nodes' centrality
	Filtering results	The brokers were selected based on the centrality values

## 4 Experimental Design, Materials and Methods

### 4.1 Soil sample collection and physicochemical property analysis

**Fig. 2:**
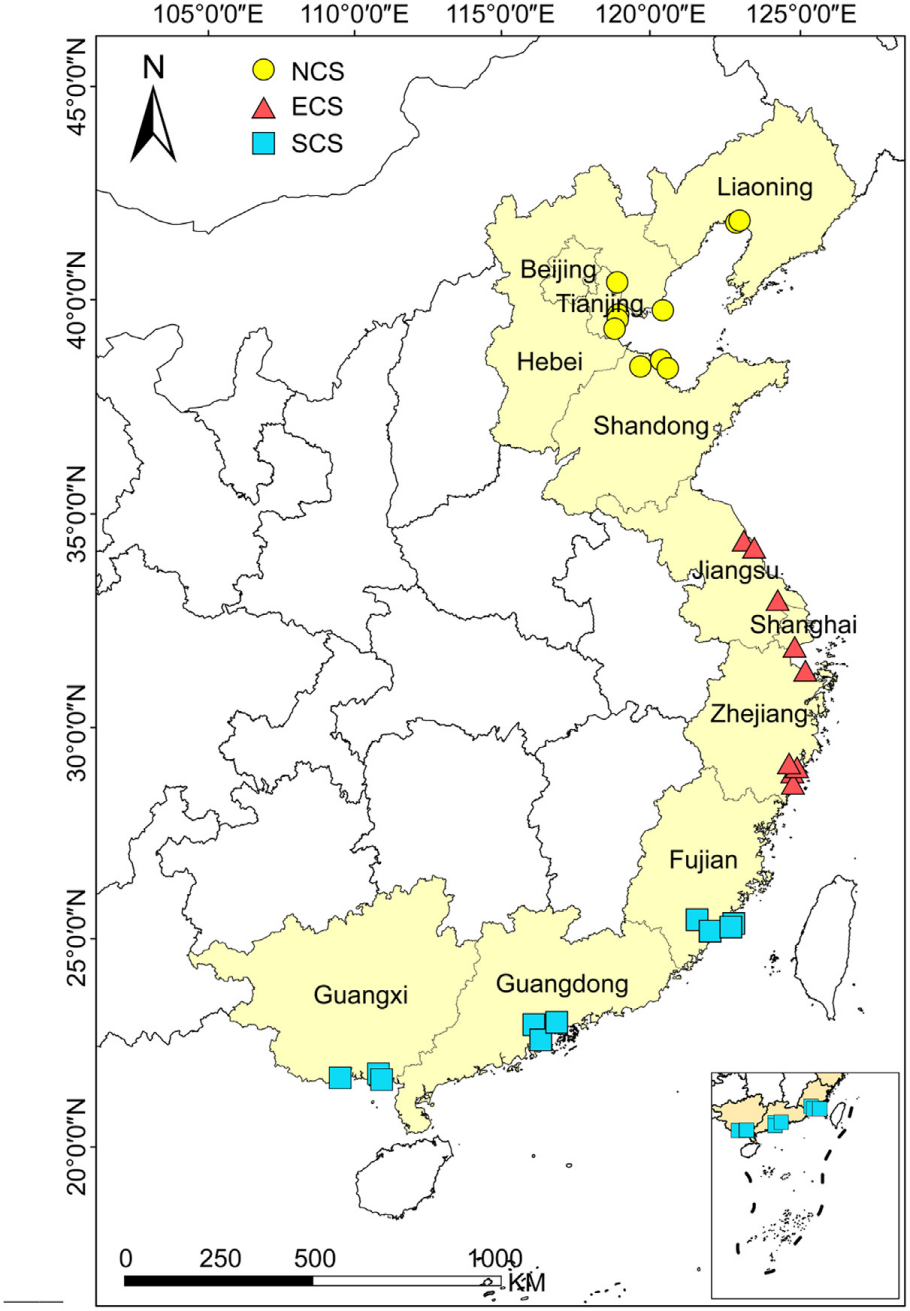
The sampling sites in China's estuarine wetlands.

Surface soil samples (0–5 cm depth) were collected from nine coastal estuaries in China during August-September 2019. Estuaries sampled included the Liaohe (LH), Haihe (HH), Yellow (YR), Sheyanghe (SYH), Changjiang (CJ), Oujiang (OJ), Jiulongjiang (JLJ), Zhujiang (ZJ) Rivers, and the Beibuwan Gulf (BBW) (Fig. 2). For each estuary, 2–4 sampling areas were established. Within each area, 15–20 individual soil samples were composited and homogenized to form a representative sample, yielding a total of 29 composite samples. Following sterilization and sealing, subsamples designated for DNA analysis were flash-frozen in liquid nitrogen, while subsamples for physicochemical analysis were stored at −20 °C. Soil physicochemical properties—including pH, moisture content (MC), dissolved oxygen (DO), salinity, organic carbon (OC), microbial oxidizable ferrous iron (Fe²⁺), reducible ferric iron (Fe³⁺), sulfide (S), and inorganic nitrogen components (exchangeable NO₂⁻-N, NO₃⁻-N, and NH₄⁺-N)—were measured using standard methods as previously described [5].

### 4.2 DNA extraction and high-throughput sequencing

Total genomic DNA was extracted from soil samples using the FastDNA SPIN Kit for Soil (MP Biomedicals, Solon, OH, USA). Bacterial and fungal communities were characterized by amplifying the 16S rRNA V3-V4 region (primers 338F/806R) and the internal transcribed spacer 1 (ITS1) region (primers ITS1737/ITS2043), respectively [6]. For each sample, PCR amplicons generated from three independent amplification reactions were pooled. Amplicons were verified via electrophoresis (1.5 % agarose gel), and target bands were excised and purified using the E.Z.N.A.® Gel Extraction Kit (Omega Bio-tek, Norcross, GA, USA). Sequencing libraries were constructed following standard protocols with the NEXTflex™ Rapid DNA-Seq Kit (Bioo Scientific, Austin, TX, USA). Paired-end sequencing (2 × 250 bp) was performed on an Illumina MiSeq platform by Shanghai Meiji Gene Biotechnology Co., Ltd. Raw paired-end reads were merged and quality-filtered using FLASQ (v0.14.1). Operational Taxonomic Units (OTUs) were clustered at 97 % sequence similarity using UPARSE. Representative OTU sequences were taxonomically classified against the SILVA (bacteria) and UNITE (fungi) databases [7]. Raw sequence data are deposited in the NCBI Sequence Read Archive (SRA) under BioProject accession number PRJNA755846.

### 4.3 Construction of bacterial and fungal MEN

**Fig. 3:**
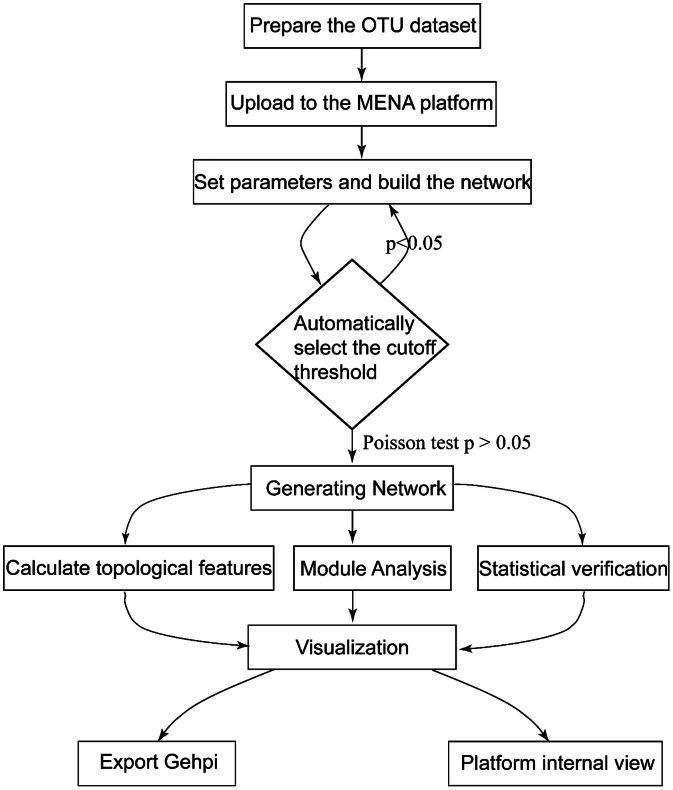
Flowchart for constructing MEN using the MENAP platform and Gephi software.

**Fig. 4:**
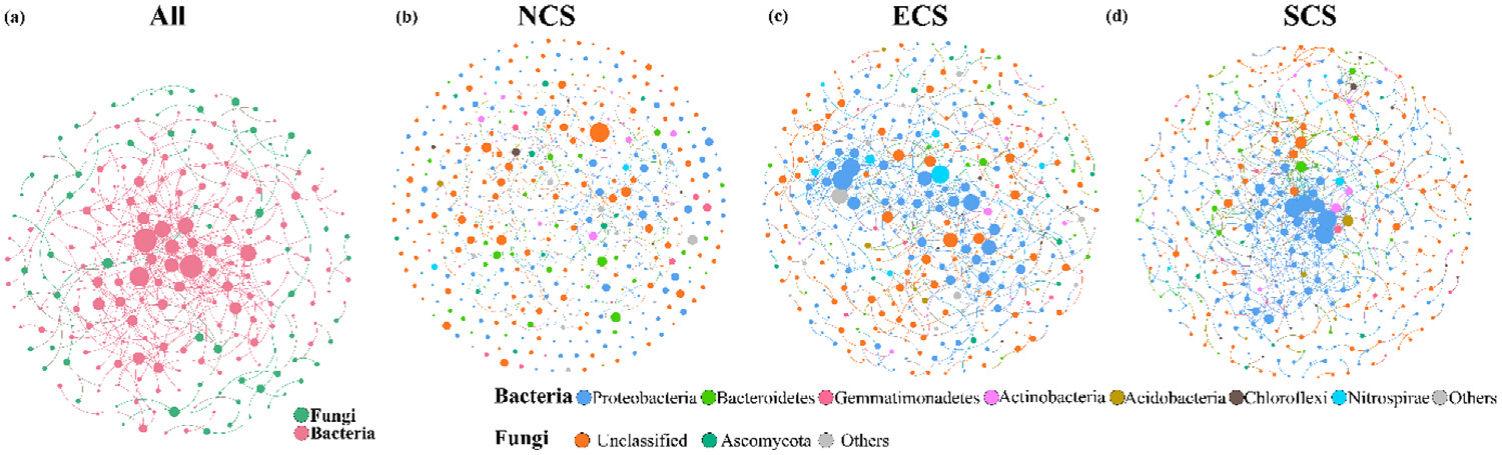
The result graph of MEN construction using the MENAP platform and Gephi software.

Molecular ecological networks (MENs) were constructed using the Molecular Ecological Network Analysis Pipeline (MENAP; http://ieg2.ou.edu/MENA/) following its standard workflow [8] (Fig. 3). Briefly, the filtered OTU abundance table was uploaded to the MENAP platform. Pearson correlation matrices were calculated based on log-transformed OTU abundances, including only OTUs detected in more than half of the soil samples to ensure robustness. The platform automatically determined the correlation significance threshold (St) for adjacency matrix construction using random matrix theory (RMT), selecting the optimal cutoff where the nearest-neighbor spacing distribution of eigenvalues conformed to the Poisson distribution (*p* > 0.05). An adjacency matrix was generated, retaining only statistically significant correlations where the absolute value of the coefficient was ≥ St. Topological features (e.g., connectivity, centrality) were calculated, and modules were identified within the platform. Statistical verification of key network properties was performed. The resulting networks were visualized and refined using Gephi 0.9.2, with node colors representing either taxonomic classifications at the phylum level (e.g., Proteobacteria, Ascomycota), and node sizes scaled according to topological indices (e.g., degree centrality). Final visualizations were exported from Gephi (Fig. 4).

### 4.4 Statistical analysis

**Fig. 5:**
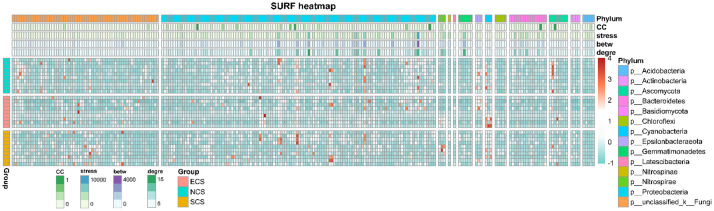
The result graph of ComplexHeatmap.

Topological Feature Heatmap: A comprehensive heatmap integrating taxonomic classification, relative abundance, and topological indices of network nodes was generated using the R package ComplexHeatmap [9]. OTU abundance data underwent sequential preprocessing: 1) standardization (z-score transformation) using the decostand() function (vegan package), 2) log₂(*x* + 1) transformation to normalize variance. Row annotations incorporated taxonomic classifications at the phylum level, while column annotations represented sample groups. Heatmap color gradients were defined using colorRampPalette() with customized breakpoints, and clustering was disabled to maintain predefined sample/OTU ordering. Final visualizations were exported in vector format (Fig. 5). *Broker Node Identification:* Key connector nodes ("brokers'') linking distinct network modules were identified through centrality analysis. Nodes were ranked by degree, stress, betweenness, and Clustering Coefficient [10]. (Table 2).

**Table 2:** “Broker” properties of the global MEN.

OTU-ID	Node degree	Node betweenness	Node stress	Node Clustering Coefficient
BAC832	4.00	992.04	2279.00	0.33
BAC19425	10.00	864.64	3715.00	0.20
BAC18509	10.00	2144.18	5249.00	0.16
BAC17093	11.00	1593.12	4334.00	0.13
BAC16524	18.00	1921.36	7319.00	0.14
BAC14925	13.00	1348.17	4902.00	0.22
BAC1480	7.00	1184.35	3571.00	0.19
BAC14399	10.00	1115.11	5131.00	0.00
BAC14340	11.00	1300.23	4142.00	0.04
BAC12944	12.00	804.38	3523.00	0.15
BAC11551	11.00	878.93	3772.00	0.07
BAC11548	15.00	4039.16	10673.00	0.07
BAC5565	11.00	419.28	1832.00	0.29
BAC6948	15.00	1752.18	5863.00	0.17
BAC871	10.00	973.33	5002.00	0.11

The specific R code for implementing the above topological feature heat map construction and intermediate node identification is as follows:

**# 1. Clear environment variables**

rm(list=ls())

**# 2. Load the R packages required for analysis**

library(pheatmap) # heatmap drawing

library(vegan) # data standardization

library(ggplot2) # fundamentals of visualization

library(ggh4x) # graphic expansion function

library(dplyr) # data processing

library(RColorBrewer) # color schemes

**# 3. Set the working directory**

setwd("C:/XXX/XXX'')

**# 4. Read the expression matrix data (row names as features and column names as samples)**

test <- read.table("all.txt'', sep="\t'', header=TRUE, row.names=1)

**# 5. Preliminary heat map visualization and data distribution exploration**

pheatmap(test)

pheatmap(test, display_numbers = TRUE)

pheatmap(test, display_numbers = TRUE, number_format = ``" %.1e'')

range(test)

**# 6. Perform horizontal normalization (Z-score transformation) to eliminate the magnitude differences between features**

test.1 <- decostand(test, ``standardize'', MARGIN = 1)

apply(test.1, 1, sd) # Verify the effect of standardization

range(test.1)

**# 7. log2 transformation (+1 to avoid the influence of zero values) compresses the dynamic range of the data**

test.2 <- log2(test.1 + 1)

range(test.2)

**# 8. Read the sample (column) and feature (row) annotation information**

annotation_col <- read.table("annotation_col.txt'', sep="\t'', header=TRUE, row.names=1)

annotation_row <- read.table("annotation_row.txt'', sep="\t'', header=TRUE, row.names=1)

**# 9. Heat map optimization: Integrate annotation information and disable clustering to maintain the original order of the data**

pheatmap(test.1, annotation_row = annotation_row)

pheatmap(test.1, annotation_col = annotation_col, annotation_row = annotation_row,

cluster_row = FALSE, cluster_cols = FALSE)

**# 10. Customize the heat map color scheme and group divider lines**

pheatmap(test.1, annotation_col = annotation_col, annotation_row = annotation_row,

gaps_row = *c*(29), cluster_row = FALSE, cluster_cols = FALSE,

color = colorRampPalette(c("white'', "red''))(10,000))

**# 11. Define the annotation color scheme**

ann_colors = list(group = *c*(High = "#E54B34'', Low = "#4CBAD4''))

**# 12. Set a color gradient breakpoint**

bk = unique(c(seq(−2, 5, length=100)))

range(test.2)

**# 13. Draw the final heat map (hide the row and column names to simplify the view, adjust the cell size)**

*p* <- pheatmap(test.2, annotation_col = annotation_col, annotation_row = annotation_row,

gaps_row = *c*(10, 19), gaps_col = *c*(63, 181, 184, 185, 186, 192, 195, 198, 203, 219, 227, 231),

cluster_row = FALSE, cluster_cols = FALSE,

color = colorRampPalette(c("#79CDCD'', "#FAFAFA'', "white'', "#FF7F50'', "firebrick''))(100),

show_colnames = FALSE, show_rownames = FALSE,

cellwidth = 3.3, cellheight = 5, main = "SURF heatmap'')

**# 14. Save the heat map in SVG format**

ggsave(p, file = "heatmap.svg'', width = 30, height = 15)

## Limitations

None.

## Ethics Statement

No animal or human experiments were performed in this study.

## Credit Author Statement

**Yingjie Ma:** Data Curation, Writing-Original Draft, Visualization; **Shuhan Wang:** Methodology, Software; **Jiale Ding:** Validation and Data Curation; **Yingpai Liu:** Validation and Data Curation; **Yonghui Wang:** Visualization, Review & Editing; **Zongxiao Zhang:** Supervision, Review & Editing.

## Data Availability

Data availability:Dataset on soil microbial community composition based on 16S rRNA gene OTUs from estuarine wetlands in China (Original data).

## References

[1] Dai T., Zhang Y., Ning D. (2018). Dynamics of sediment microbial functional capacity and community interaction networks in an urbanized coastal estuary. Front. Microbiol..

[2] Álvarez-Barragán J., Cravo-Laureau C., Duran R. (2022). Fungal-bacterial network in PAH–contaminated coastal marine sediment. Environ. Sci. Pollut. Res..

[3] Ji W., Zhou Z., Yang J. (2025). Soil bacterial community characteristics and functional analysis of estuarine wetlands and nearshore estuarine wetlands in Qinghai Lake. Microorganisms..

[4] Zhang J., Ding X., Guan R. (2018). Evaluation of different 16S rRNA gene V regions for exploring bacterial diversity in a eutrophic freshwater lake. Sci. Total Environ..

[5] Zhang Z., Han P., Zheng Y. (2023). Spatiotemporal dynamics of bacterial taxonomic and functional profiles in estuarine intertidal soils of China coastal zone. Microb. Ecol..

[6] Corcoll N., Österlund T., Sinclair L. (2017). Comparison of four DNA extraction methods for comprehensive assessment of 16S rRNA bacterial diversity in marine biofilms using high-throughput sequencing. FEMS Microbiol. Lett..

[7] Edgar R.C (2018). Updating the 97% identity threshold for 16S ribosomal RNA OTUs. Bioinformatics..

[8] Deng Y., Jiang Y.H., Yang Y. (2012). Molecular ecological network analyses. BMC. Bioinformatics..

[9] Gu Z. (2022). Complex heatmap visualization. Imeta.

[10] Von Schiller D., Acuña V., Aristi I. (2017). River ecosystem processes: a synthesis of approaches, criteria of use and sensitivity to environmental stressors. Sci. Total Environ..

